# Functional Disability Among Middle-Aged and Older Adults in China: The Intersecting Roles of Ethnicity, Social Class, and Urban/Rural Residency

**DOI:** 10.1177/00914150221092129

**Published:** 2022-04-14

**Authors:** Shen (Lamson) Lin

**Affiliations:** Factor-Inwentash Faculty of Social Work, 152790 University of Toronto, Toronto, Canada

**Keywords:** intersectionality, social determinants of health, IADL disability, health inequity, ‌minority aging

## Abstract

This study explores how ethnicity, family income, and education level differentiate patterns of functional limitations among urban and rural Chinese (aged 45 ≥ years). Based on the 2018 China Family Panel Studies (CFPS) (n = 16,589), this nationwide study employed binary/multinomial logistic regression analyses, stratified by urban/rural residency, to estimate the likelihood of instrumental activities of daily living (IADLs) disability (0/1–2/≥3 limitations) by social determinants of health (SDoH). The estimated overall prevalence of IADLs disability was 14.3%. The multivariable analyses did not find significant ethnic disparity in IADLs disability in urban China, while in rural China, ethnic minorities were 44% more likely to have IADLs disability than Han Chinese. Among rural residents, Mongolians, Tibetans, and Yi minority more than tripled the odds of having ≥3 limitations than Han Chinese; and the intersections of ethnicity and social class were associated with functional limitations. Long-term care and anti-poverty programs should target minority aging populations in rural China.

## Introduction

With more than 1.4 billion people, China, as the world's most populous country, has experienced an increasingly widening trend of socioeconomic and urban–rural inequality in the aftermath of 1980s market-oriented economic reform (Xie & Zhou, 2014). There have been widespread concerns about these social stratifications resulting in remarkable health inequalities in China (Meng et al., 2008; Yang et al., 2013; Zhou et al., 2019). Ethnicity is an important dimension of social stratification (Hui-Fang et al., 2019) and yet ethnic health disparity is still an under-research topic in China, when compared with its rich literature in western industrial nations (Mendes de Leon et al., 2005). The prevalence of physical disability is a key marker of population health and total morbidity burden (Gjonça et al., 2009), which parallels with the human aging process and lifespan extension, due to decreased skeletal muscle mass, cognitive impairments, and noncommunicable diseases (Feng et al., 2020). Thus, extending disability-free life expectancy for the aging population is a crucial public health objective in China and across the globe (Prina et al., 2020).

## Disability, Ethnicity, and Intersectionality

Since the 1960s, theories of disability have been constantly evolving (Purser et al., 2012; Verbrugge & Jette, 1994). Functional disability is increasingly recognized as a multifaceted and dynamic process that includes biological conditions but also social and environmental contexts (Liang et al., 2017). Countering pure biomedical frameworks, the sociomedical model of disablement argues that upstream social determinants are fundamental in shaping older adults’ opportunity to access broadly serviceable recourses that could perpetuate health inequities in the disability of performing socially defined roles and activities (Gjonça et al., 2009; Phelan & Link, 2005; Verbrugge & Jette, 1994). The framework of the World Health Organization (WHO) commission on social determinants of health (SDoH) asserts that social vulnerabilities, produced by one's multiple social locations in hierarchical social orders, are root causes of population health inequalities in the upstream causal pathway to ill-health (Lin, 2021; WHO, 2008). In the case of old-age disability, the distal impact of social vulnerabilities (e.g., poverty, racial stratification) could be transmitted through many downstream channels (Palmer et al., 2019). For instance, older adults with inadequate access to health care had considerably greater odds of instrumental activities of daily living (IADL) disabilities in comparison to their peers with sufficient access to health care in both rural and urban China (Zhang et al., 2017). Uninsured older Chinese who were previously unemployed had worsened physical functions than their insured counterparts (Liu et al., 2016).

China is known to be a multiethnic country with 56 ethnic groups: the Han ethnicity makes up the majority of the population, accounting for 92% of the total, while the remaining 55 ethnic groups are officially classified as Chinese ethnic minorities (Hui-Fang et al., 2019). Although scientists have been exploring the scale, the trends, and the mechanisms of old-age disability in China (Feng et al., 2013; Liang et al., 2017; Liu et al., 2020; Tang et al., 1999; Yan et al., 2019), no empirical studies have systematically analyzed ethnic stratification in old-age disability in China to date. Within the limited evidence landscape, prior literature has found that ethnic minority older adults had significantly lower scores of physical functions than the Han Chinese majority, but these findings were solely based on simplistic comparisons of bivariate and correlation analysis without controlling for many potential confounders (Matsubayashi et al., 2009; Ran, Jiang et al., 2017; Ran, Kong et al., 2019). Some investigations treated race/ethnicity as a covariate (Liang et al., 2017; Ma et al., 2017), while others focused on within-group differences among ethnic minorities and failed to compare with the Han Chinese majority (Li et al., 2007). Even when ethnic disparities were reported, the conventional approach tends to treat ethnic minorities as a homogenous group (Gu & Xu, 2007; Li et al., 2007; Ouyang & Pinstrup-Andersen, 2012; Xu et al., 2020) while neglecting the heterogeneity and intra-group differences among Chinese ethnic minorities with diverse cultural, geographical, and linguistic backgrounds. This aggregated category of ethnic minority may conceal underlying health inequities between different ethnic subgroups.

On a global scale, although functional health inequalities are well documented in relation to race/ethnicity (Mendes de Leon et al., 2005), income (Beydoun & Popkin, 2005; Brenner & Clarke, 2018), and education (Liu et al., 2013), prior research has typically examined these health differences separately. The intersectionality lens of ethnicity and social class has been largely overlooked (Gkiouleka et al., 2018). As such, intersectionality theory has much to offer to aging research because it unpacks various minority struggles that are often obscured in the traditional approach (Kapilashrami & Hankivsky, 2018). While a growing body of western literature has adopted the intersectionality framework to understand health inequalities (Roy et al., 2020; Warner & Brown, 2011), little is known about the utility of this theory in the Chinese context. Transferring the intersectionality lens to the Chinese populations could illuminate the compounding effects of multiple inequities to understand the experience of systemic oppression among older persons in non-Western societies.

## Research Objectives and Hypothesis

The present population-based study seeks to address the aforementioned research gaps and extends the existing literature on Chinese older adults by (a) adopting a disaggregated approach of diverse ethnic minority groups relative to the Han Chinese majority and (b) and by quantifying the intersectionality lens of multiple social vulnerabilities with respect to ethnicity, income, and education to understand how the clustering of social inequalities combine to shape functional health disparities. According to the 2010 census (Feng et al., 2020), the number of older adults (aged 60 years) with disabilities in China is estimated to reach 42.7 million (24%). Since China is working toward reducing rural–urban inequalities in the health care system and developing long-term care (LTC) insurance for the aging populations, it is critical to identify whether and to what extent LTC needs arise from functional health disparities in ethnicity, social class, and geographic location. Ethnic minorities in China, like those in many other countries, often encounter similar social disadvantages, such as living in rural remote regions, having limited socioeconomic resources, and being underserved by the health care system compared with the majority population, all of which are known risk factors for adverse health outcomes, including disability (Hui-Fang et al., 2019; Ran, Kong et al., 2019).

Informed by the SDoH framework (WHO, 2008) and intersectionality lens (Kapilashrami & Hankivsky, 2018), this study aimed to explore differential patterns of early stage functional loss—disability in IADLs—by diverse Chinese ethnicity, family income, education, the intersecting profile of these three social positionings, and how these patterns differ by rural/urban residency among Chinese individuals aged 45 years and above. It could serve as a timely reference to understand ethnic-specific differences in early physical dysfunction and could inform appropriate evidence-based policies for planning LTC services and home care programs in the community (Feng et al., 2020; Zhu & Österle, 2017). Recognizing that minority communities with adverse social determinants may be more susceptible to the old-age disability, the present study examined the following four hypotheses:

H1 Ethnic inequalities hypothesis: Persons who are ethnic minority Chinese would be more likely to have functional limitations compared to the Han majority Chinese.
H2 Socioeconomic inequalities hypothesis: Persons who are less educated and/or low-income would be more likely to have functional limitations compared to their peers in higher social class.
H3 Urban–rural stratification hypothesis: The effect of disadvantaged social positions in terms of ethnicity, income, and education on functional limitations depends on the urban–rural residency.
H4 Intersecting inequalities hypothesis: Multiple jeopardies of disadvantaged social positions in terms of income, education, and ethnicity status would put older adults at a higher risk of having functional limitations compared to their peers with relative social privilege.

## Methods

### Data Sources and Study Population

This study analyzed the 2018 cross-sectional data (i.e., personal dataset linked with the family economy dataset) of the China Family Panel Studies (CFPS), an ongoing nationwide longitudinal survey project launched by the China Social Sciences Research Centre at Peking University in 2010 (Lin, 2020). The CFPS covers 25 of 31 provinces and/or ethnic minority autonomous regions in China and excludes Hong Kong, Macao, and Taiwan, Xinjiang, Tibet, Qinghai, Inner Mongolia, Ningxia, and Hainan and the detailed methodology and sampling design have been previously published (Xie & Lu, 2015). Using a multistage to size (PPS) strategy, the CFPS 2018 survey began from June 2018 to May 2019 and was collected through in-home face-to-face interviews (78%) or telephone interviews (22%) with a total sample of 32,669 valid individuals (responsive rate: 67.4%). In this study, participants were excluded if they aged less than 45 years old or did not respond to all questions related to the analysis. This yielded a final sample size of 16,661 respondents. Institutional Research Ethics Board review was not required for the research involving secondary use of publicly available CFPS anonymized data classified as nonhuman subjects.

### Study Variables

#### Disability in Instrumental Activities of Daily Living

The CFPS measured IADLs for respondents aged 45 years and above and it is a modified version of the widely used Lawton IADLs scale by replacing communication, medication, and finance management with two basic skills (i.e., ambulating and feeding) from activities of daily living (ADLs) scale (Lawton & Brody, 1969). The CFPS's IADLs scale assesses the ability to perform self-care activities to live independently in the community (Feng, 2018). Respondents were asked if they could perform these seven tasks without assistance: (1) outdoor activities (e.g., walking 300 m); (2) kitchen chores (e.g., preparing meals and washing dishes); (3) shopping for groceries; (4) having meals; (5) managing transportation; (6) housekeeping (cleaning); and (7) doing laundry. These seven yes/no items were then summed into a count of IADLs disabilities (range: 0–7). Consistent with prior literature (He et al., 2019), two aggregated variables were generated: (1) a binary measure (none; ≥1 limitation) and (2) a three-level measure (none; moderate: 1–2 limitations; and moderately severe: ≥ 3 limitations). The report of three or more IADLs limitations was of particular interest because this level of limitation is often required by LTC insurance policies for individuals to qualify for benefits in China. Compared to basic ADLs, IADLs examine more sophisticated self-maintaining tasks (e.g., complex thinking skills, organizational skills) and require higher neuropsychological functioning, which can be significantly hampered in persons with mild cognitive impairment (Liang et al., 2017). The IADLs differ from the ADLs in that, when these tasks become challenging to do independently, persons began to seek outside assistance from individuals and/or mechanical devices. Hence, as an early sign of functional decline in old age, measurement of IADL functions is essential because these are predictors of the need for alternative living arrangements, the usage of paid home care, and admission to nursing homes.

#### Social Determinants of Health

In light of the SDoH paradigm (Lin, 2021; WHO, 2008), ethnicity status, income, education levels, and residential status were selected as four key equity stratifiers in this study. *Ethnicity* was first operationalized as an aggregated variable (Han vs. non-Han Chinese); for subsequent analyses, it was then decomposed into eight groups: 1 = the Han majority; 2 = Mongolians; 3 = Tibetans; 4 = Miao; 5 = Yi; 6 = Buyi; 7 = Manchu/Manzu; 8 = other ethnic minorities (e.g., Dong/Gaeml, Yao, and Bai). *Education* was measured by the highest degree attained and was divided into four categories: 1 = less than grade six; 2 = primary school graduate; 3 = junior high school graduate; 4 = senior high school and above. *Household income* was classified according to the percentile of household income per capita in the past 12 months (< ¥8k; ¥8k–<¥15.4k; ¥15.4k–<¥29.7k; and ≥¥29.7k). *Urban/rural residency* was classified by the unique household (“*hukou”*) registration system that identifies a person as a permanent resident of a certain location based on agricultural (rural) and nonagricultural (urban) status in China, consistent with prior literature (Song & Smith, 2019). The residential status was a potential effect modifier since it was historically linked to the urban–rural divide of opportunity structures, social benefit programs, and welfare entitlement (Yu et al., 2020), with rural residents (i.e., rural hukou holders) being systemically marginalized, than urban residents, in almost every aspect of life such as educational stratification (Wu, 2011), suboptimal coverage of health insurance (Lin, 2020).

#### Intersecting Social Vulnerability

Informed by the intersectionality lens to examine multiple forms of marginalization (Kapilashrami & Hankivsky, 2018), a cumulative profile of social vulnerabilities was created to identify respondents in one or more out of three disadvantaged social positions: ethnic minorities status, living in poor households at the lowest income strata (<¥8k/per year), being at lowest education level (<grade 6).

#### Covariates

To consider other potential confounders known to be involved in the aging process, control variables include social demographics (age groups, sex, marital status, retirement status), health indicators (self-rated health, depressive symptoms), health care utilization (hospital visit), and access to social welfare (health insurance and social benefit). *Depressive symptoms* were measured by the Chinese version of the 8-item Center for Epidemiologic Studies – Depression scale (CESD-8). The Chinese version of the CES-D scale has demonstrated good reliability and validity in the Chinese aging population (Djundeva et al., 2018). The CESD-8 composite measure (range: 0–24) was a 4-point Likert scale, from 0 (rarely: <1 day) to 3 (almost every day: 5–7 days), to assess the frequency of the feelings of hopelessness, restlessness, depression, worthlessness, and everything being an effort in the past 7 days. A cut-off score of 5 out of 24 was applied to screen out those who are at risk of having depressive symptoms. *Social benefit* was a binary variable (yes/no) indicating whether the household has received any kind of social assistance in the past 12 months, such as minimum living standard guarantee (the “Dibao”) program. The Dibao policy is the largest social safety net in China, which attempts to alleviate poverty through mean-tested conditional cash transfers to households whose income per capita is below the poverty lines as defined by the local governments (Evandrou et al., 2014).

### Statistical Analysis

First, chi-square tests were used to test the statistical differences at the bivariate level. Second, to test Hypotheses 1 and 2, binary logistic regression models were used to examine between association three key social determinants (i.e., ethnicity, income, and educational levels) and the likelihood of having IADLs limitations (yes/no); to test Hypothesis 3, multiplicative interactions were examined by including the product terms between residency (urban/rural) and three key social determinants. A sensitivity test was conducted for two samples stratified by residency as the interaction terms were significant. Third, to examine the severity of IADLs limitations and the subgroups of ethnic minorities, multinomial logistic regression models were undertaken to calculate the odds ratio (OR) of having 1 to 2 and ≥ 3 IADLs limitations for three key social determinants, after controlling for other potential confounders. Lastly, to test Hypothesis 4, another multinomial logistic regression analysis was conducted for the same outcome measure with the cumulative profile of social vulnerabilities as the key explanatory variable, adjusted for covariates. Statistical analyses and data management were performed using the SPSS software package, Version 26 (IBM Corp., Armonk, NY, USA). A *p* value <.05 (two-tailed) was considered statistically significant. Model performance was assessed by the Nagelkerke R^2^ statistic (a measure of the proportion of explained variation in the logistic model).

## Results

### Sample Characteristics

Table 1 summarizes the sample characteristics for all variables stratified by urban/rural residency and ethnicity status. The whole sample (n = 16,589) mainly consisted of respondents who were Han Chinese (92.7%), aged 45–76 years (82.5%), junior high school graduate or below (77.8%), married (87.6%), and had health insurance (93.6%). The sex distribution was even (men: 49.7%; women: 50.3%). Around half (48.1%) of the sample received social benefits in the past 12 months. In terms of health conditions, around a quarter reported poor self-rated health (24.3%) and about one-third screened positive for CESD-8 depression (30.6%). One in seven (14.2%) of the sample had at least one IADLs limitation and the prevalence of having ≥ 3 IADLs limitations was 4.2%. Of the seven items for IADLs, the most reported limitation was managing transportation (9.2%), whereas the least reported item was having a meal (0.9%).

**Table 1.** Demographic Characteristics Stratified by Residency-by-Ethnicity Status, in the China Family Panel Studies (CFPS) 2018, Persons Aged ≥ 45 Years.

	Total	Urban residents			Rural residents		
		Han	Minority	*P* value	Han	Minority	*P* value
Sample size	16,589	7,557	370		7,822	840	
**Ethnicity**							
Han	92.7%	–	–	–	–	–	–
Minority	7.3%	–	–		–	–	
**Income (quantile)**				**<.001**			**<.001**
< ¥8k	25.0%	12.8%	23.2%		34.8%	44.8%	
¥8k–<¥15.4k	25.0%	18.5%	25.1%		30.6%	30.8%	
¥15.4k–<¥29.7k	25.0%	28.1%	25.7%		22.9%	17.1%	
≥¥29.7	25.0%	40.7%	25.9%		11.7%	7.3%	
**Education**				**<.001**			**<.001**
<grade 6	35.3%	25.6%	39.2%		42.4%	54.8%	
Primary school	24.8%	22.0%	19.5%		28.1%	22.4%	
Junior high school	25.0%	29.4%	24.9%		21.7%	16.8%	
≥Senior high school	14.9%	23.1%	16.5%		7.8%	6.1%	
**Covariates**							
**Age**				.238			.078
45–54	38.6%	38.1%	42.2%		38.7%	41.3%	
55–65	31.0%	30.8%	29.2%		31.1%	32.4%	
65–74	22.9%	22.7%	19.2%		23.6%	19.6%	
≥75	7.5%	8.4%	9.5%		6.7%	6.7%	
**Sex**				**.023**			.904
Male	49.7%	49.0%	43.0%		50.6%	50.4%	
Female	50.3%	51.0%	57.0%		49.4%	49.6%	
**Retirement**				.429			.629
No	68.5%	56.3%	58.4%		79.5%	80.2%	
Yes	31.5%	43.7%	41.6%		20.5%	19.8%	
**Marriage**				**.037**			**<.001**
Single/widow	12.4%	12.3%	15.9%		11.8%	17.9%	
Married	87.6%	87.7%	84.1%		88.2%	82.1%	
**CESD-8 Depression**				**.003**			.867
Negative	69.4%	75.0%	68.1%		64.5%	64.8%	
Positive	30.6%	25.0%	31.9%		35.5%	35.2%	
**Self-rated health**				**.004**			.579
Good/fair	75.7.%	79.7%	73.5%		72.2%	73.1%	
Poor	24.3%	20.3%	26.5%		27.8%	26.9%	
**Hospitalization**				**.011**			**.014**
No	81.8%	82.2%	77.0%		81.9%	78.5%	
Yes (past year)	18.2%	17.8%	23.0%		18.1%	21.5%	
**Health insurance**				.381			.871
Insured	93.6%	93.1%	91.9%		94.1%	93.9%	
Uninsured	6.4%	6.9%	8.1%		5.9%	6.1%	
**Social benefit**				**<.001**			**<.001**
Yes (past year)	48.1%	30.1%	43.2%		63.3%	70.1%	
No	51.9%	69.9%	56.8%		36.7%	29.9%	
**IADLs limitations**							
0 limitation	85.8%	89.1%	86.8%	.362	83.4%	77.5%	**<.001**
1–2 limitations	10.0%	7.5%	9.2%		11.9%	14.9%	
≥ 3 limitations	4.2%	3.4%	4.1%		4.7%	7.6%	
**Specified IADLs Limitations**							
Outdoor activities	4.9%	3.8%	4.3%	.596	5.7%	8.3%	**.002**
Kitchen chores	4.0%	3.3%	4.9%	.438	4.5%	5.5%	.219
Shopping	5.4%	4.1%	5.9%	.214	6.3%	9.0%	**.002**
Having meals	0.9%	0.6%	0.5%	.869	1.1%	1.4%	.371
Transportation	9.2%	7.3%	8.4%	.713	10.5%	14.9%	**<.001**
House cleaning	3.2%	2.9%	3.2%	.889	3.4%	5.0%	**.014**
Laundry	4.4%	3.5%	4.6%	.495	4.8%	8.2%	**<.001**

*Note:* CESD-8 = Chinese version of the 8-item Center for Epidemiologic Studies – Depression scale; IADL = instrumental activities of daily living

Compared to the Han majority, the ethnic minority was overrepresented in the lowest income strata (< ¥8k/year; urban sample: 23.2% vs. 12.8%; rural sample: 44.8% vs. 34.8%), at the bottom of the education gradient (<grade 6; urban sample: 39.2% vs. 25.6%; rural sample: 54.8% vs. 42.4%) and more likely to be single/windowed and had past-year hospitalization, regardless of urban/rural residency. However, the ethnic difference in IADLs limitations was only found significant among the rural population, with ethnic minority bearing greater burden of IADLs disability than the Han majority Chinese (≥ 3 limitations: 7.6% vs. 4.7%, *p* < 0.001), while no ethnic disparities in IADLs disability were found for the urban sample (*p* = .362). Task-specific comparisons further indicate that significant Han-minority differences were observed in most IADLs tasks other than eating and preparing meals in rural areas. All *a priori* covariates were significantly linked to IADLs limitations at the bivariate level and thus no variable was excluded in the multivariable analyses to achieve a parsimonious model.

### Multivariable Logistic Regression

Table 2 shows the results of the binary logistic regression analysis to test the interaction terms using four separate models. Compared to the original Model 1, the interaction terms in Model 2 were found statistically significant for ethnicity and residency; thus, two separate analyses were replicated for urban (Model 3) and rural communities (Model 4), respectively. Notably, the Nagelkerke R^2^ statistics indicate that the established binary logistic model could explain 24.6% variance of IADLs limitations for the overall sample and 22.4% variance for the rural sample. Focusing on the rural sample, Table 3 shows the results of multinominal logistic regression analysis to illustrate the overall counts of sample characteristics, prevalence, and adjusted odds of having one and/or two (1 to 2) and multiple (≥3) IADLs limitations (Nagelkerke R^2^ = 25.2%). As shown in Table 3, among the rural sample, older age (ORs = 1.51–5.00, *p* < .001), being male (OR = 1.76, 95% *CI*: 1.38–2.44), retirement (OR = 6.86, 95% *CI*:5.32–8.84), having screen-positive CES-D depressive symptoms (OR = 1.86, 95% *CI*: 1.47–2.35), and poor self-rated health (OR = 4.50, 95% *CI*: 3.51–5.76) were linked to an increased likelihood of having multiple (≥3) IADLs limitations.

**Table 2.** Adjusted Odds Ratios (OR) of IADLs Limitations (yes/no) in the CFPS 2018, Persons Aged ≥ 45 Years.

	Model 1 Whole sample (N = 16,589)			Model 2 Whole sample (N = 16,589)			Model 3 Urban residents (N = 7,927)			Model 4 Rural residents (N = 8,662)		
	R^2^ = 24.6%			R^2^ = 24.7%			R^2^ = 26.4%			R^2^ = 22.4%		
	OR	95% *CI*		OR	95% *CI*		OR	95% *CI*		OR	95% *CI*	
**Independent variables**												
** Ethnic minority (Ref. Han Chinese)**	1.30**	**1.10**	**1.54**	1.45***	**1.20**	**1.76**	0.96	0.68	1.35	1.44***	**1.19**	**1.75**
** Income (Ref. ≥ ¥29.7k)**												
< ¥8k	1.63***	**1.39**	**1.91**	1.60***	**1.25**	**2.04**	1.53***	**1.21**	**1.94**	1.65***	**1.30**	**2.11**
¥8k–<¥15.4k	1.34***	**1.14**	**1.58**	1.30*	**1.01**	**1.68**	1.33*	**1.06**	**1.67**	1.32*	**1.03**	**1.70**
¥15.4k–<¥29.7k	1.08	0.92	1.28	1.04	0.80	1.36	1.10	0.89	1.36	1.05	0.80	1.37
** Education (Ref. ≥ Senior high school)**												
< Grade 6	2.50***	**2.05**	**3.06**	1.95***	**1.45**	**2.62**	2.79***	**2.11**	**3.69**	2.01***	**1.49**	**2.72**
Primary school	1.56***	**1.27**	**1.93**	1.21	0.89	1.65	1.80***	**1.34**	**2.41**	1.25	0.91	1.70
Junior high school	**1.22**	**0.98**	**1.51**	1.01	0.73	1.39	1.36*	**1.02**	**1.82**	1.01	0.73	1.39
**Urban residency (Ref. Rural)**	0.75***	**0.67**	**0.83**	0.79	0.44	1.40						
												
**Interaction terms**												
** Ethnicity **× **Residency**				0.66*	**0.45**	**0.97**						
** Income** × **Residency**												
< ¥8k* Res				0.99	0.71	1.38						
¥8k–<¥15.4k* Res				1.03	0.74	1.44						
¥15.4k–<¥29.7k* Res				1.05	0.75	1.47						
** Education **× **Residency**												
< Grade 6* Res				1.54*	**1.04**	**2.28**						
Primary school* Res				1.54*	**1.01**	**2.33**						
Junior high school* Res				1.35	0.87	2.08						
**Covariates**												
**Age (Ref. 45–54)**												
55–65	1.73***	**1.50**	**1.98**	1.71***	**1.49**	**1.96**	1.97***	**1.53**	**2.52**	1.64***	**1.39**	**1.94**
65–74	2.46***	**2.14**	**2.83**	2.45***	**2.13**	**2.82**	3.14***	**2.45**	**4.02**	2.22***	**1.87**	**2.64**
≥75	5.66***	**4.72**	**6.77**	5.61***	**4.68**	**6.72**	8.34***	**6.23**	**11.18**	4.27***	**3.36**	**5.42**
**Male (Ref. Female)**	0.95	0.85	1.05	0.94	0.85	1.04	1.00	0.85	1.18	0.91	0.80	1.04
**Retirement (Ref. Not retired)**	1.89***	**1.69**	**2.11**	1.90***	**1.70**	**2.12**	1.51***	**1.26**	**1.81**	2.22***	**1.93**	**2.56**
**Marriage (Ref. Married)**	1.09	0.96	1.25	1.09	0.96	1.25	1.25*	**1.02**	**1.53**	0.99	0.84	1.18
**Depression (Ref. Negative)**	1.57***	**1.41**	**1.74**	1.57***	**1.42**	**1.74**	1.69***	**1.43**	**2.01**	1.50***	**1.32**	**1.71**
**Poor self-rated health (Ref. Fair/good)**	2.23***	**2.01**	**2.48**	2.24***	**2.02**	**2.49**	2.40***	**2.02**	**2.85**	2.15***	**1.88**	**2.45**
**Hospital visit (Ref. No)**	1.34***	**1.19**	**1.49**	1.33***	**1.19**	**1.49**	1.61***	**1.35**	**1.92**	1.16	1.00	1.34
**Lack of health Insurance (Ref. insured)**	1.44***	**1.21**	**1.72**	1.44***	**1.21**	**1.72**	1.72***	**1.33**	**2.22**	1.27	1.00	1.60
**Social benefit (Ref. No)**	1.30***	**1.17**	**1.44**	1.30***	**1.17**	**1.44**	1.31**	**1.11**	**1.55**	1.28***	**1.12**	**1.46**

*Note:* CFPS = China Family Panel Studies; IADLs = instrumental activities of daily living scales; 95% *CI* = 95% confidence interval.

Statistics that reach the 0.05 level of significance are bolded. *P <0.05; **P <0.01; ***P <0.001.

**Table 3.** Prevalence (%) and Adjusted Odds Ratios (OR) of IADLs Limitations (Three-Level Severity) Among Rural Residents (n = 8,662) of CFPS 2018, Persons Aged ≥ 45 Years.

Variables		1 to 2 IADLs limitation (vs. 0)					≥3 IADLs limitations (vs. 0)				
		(N = 1,056)					(N = 429)				
	N	%	OR	95% *CI*		Sig.	%	OR	95% *CI*		Sig.
**Ethnicity (Ref. Han Chinese)**	7,822	11.90%					4.70%				
Mongolians	48	16.7%	2.15	0.97	4.79	0.060	8.30%	**5.36**	**1.72**	**16.71**	**0.004**
Tibetans	94	17.0%	1.80	0.99	3.27	0.053	24.50%	**6.17**	**3.23**	**11.81**	**<0.001**
Miao ethnic minority	181	14.9%	1.22	0.79	1.89	0.362	4.40%	1.31	0.60	2.85	0.493
Yi ethnic minority	118	20.3%	**1.97**	**1.22**	**3.20**	**0.006**	7.60%	**3.32**	**1.50**	**7.38**	**0.003**
Buyi ethnic minority	46	21.7%	1.29	0.62	2.71	0.495	2.20%	0.28	0.04	2.19	0.225
Manchu ethnic minority	151	12.6%	1.18	0.71	1.97	0.523	5.30%	1.13	0.50	2.58	0.764
Other ethnic minorities	202	10.4%	0.87	0.54	1.41	0.578	5.40%	0.95	0.47	1.95	0.890
**Income (Ref.≥ ¥29.7k)**	980	7.90%					2.20%				
< ¥8k	3,097	16.30%	**1.52**	**1.17**	**1.99**	**0.002**	7.70%	**1.99**	**1.22**	**3.26**	**0.006**
¥8k–<¥15.4k	2,652	11.50%	1.23	0.93	1.61	0.142	4.40%	**1.68**	**1.01**	**2.80**	**0.044**
¥15.4k–<¥29.7k	1,933	8.80%	1.02	0.77	1.37	0.871	2.70%	1.11	0.64	1.92	0.702
**Education (Ref.≥ Senior high school)**	662	6.80%					2.30%				
<Grade 6	3,780	17.80%	**1.93**	**1.38**	**2.71**	**<0.001**	7.30%	**2.14**	**1.19**	**3.85**	**0.011**
Primary school	2,385	9.10%	1.19	0.84	1.69	0.318	4.10%	1.32	0.72	2.41	0.367
Junior high school	1,835	6.60%	0.99	0.69	1.42	0.939	2.20%	1.02	0.54	1.94	0.946
**Covariates**											
**Age (Ref. 45–54)**	3,373	7.10%					1.70%				
55–65	2,703	11.90%	**1.70**	**1.41**	**2.04**	**<0.001**	3.40%	**1.51**	**1.06**	**2.16**	**0.023**
65–74	2,009	17.50%	**2.26**	**1.87**	**2.73**	**<0.001**	8.20%	**2.21**	**1.57**	**3.10**	**<0.001**
≥75	577	24.80%	**4.10**	**3.12**	**5.38**	**<0.001**	19.80%	**5.00**	**3.34**	**7.47**	**<0.001**
**Sex (Ref. Female)**	4,283	15.30%					4.90%				
Male	4,379	9.20%	**0.74**	**0.63**	**0.85**	**<0.001**	5.00%	**1.76**	**1.39**	**2.24**	**<0.001**
**Retirement (Ref. No)**	6,895	10.60%					1.80%				
Retired	1,767	18.40%	**1.43**	**1.21**	**1.69**	**<0.001**	17.40%	**6.86**	**5.32**	**8.84**	**<0.001**
**Marriage (Ref. Married)**	7,588	11.40%					4.00%				
Single/widow	1,074	17.80%	0.96	0.78	1.16	0.648	11.50%	1.07	0.81	1.41	0.646
**Depression (Ref. Negative)**	5,587	9.90%					3.20%				
Positive	3,075	16.30%	**1.44**	**1.25**	**1.66**	**<0.001**	8.10%	**1.86**	**1.47**	**2.35**	**<0.001**
**Poor self-rated health (Ref. fair/Good)**	6,261	9.70%					2.10%				
Poor	2,401	18.60%	**1.71**	**1.47**	**1.98**	**<0.001**	12.50%	**4.50**	**3.51**	**5.76**	**<0.001**
**Hospital visit (Ref. No)**	7,065	11.30%					3.70%				
Yes	1,597	16.30%	1.10	0.93	1.30	0.268	10.30%	1.27	1.00	1.61	0.053
**Health insurance (Ref. Insured)**	8,147	11.90%					4.80%				
Uninsured	515	16.90%	**1.32**	**1.02**	**1.71**	**0.035**	6.80%	1.15	0.77	1.73	0.503
**Social benefits (Ref. No)**	3,121	10.80%					4.50%				
Received	5,541	13.00%	**1.27**	**1.09**	**1.46**	**0.002**	5.20%	1.24	0.98	1.57	0.075
**Cumulative profile (Ref. No)**	3,212	6.60%					2.10%				
One Social vulnerability	3,430	12.70%	**1.37**	**1.00**	**1.88**	**0.048**	5.30%	1.63	0.95	2.80	0.074
Two Social vulnerabilities	1,773	20.30%	**1.88**	**1.11**	**3.19**	**0.019**	8.10%	1.98	0.79	4.95	0.144
Three Social vulnerabilities	247	19.40%	**2.37**	**1.24**	**4.54**	**0.009**	15.00%	**5.51**	**1.93**	**15.71**	**0.001**

*Note*: CFPS = China Family Panel Studies; IADLs = instrumental activities of daily living scales; 95% *CI* = 95% confidence interval. Ethnicity, education, and income were tested in one multinominal logistic regression (Nagelkerke R^2^ = 25.2%). Cumulative profile was tested in a separated identical analysis adjusted for the same covariates (not shown; Nagelkerke R^2^ = 24.8%). Cumulative profile = a sum of three social positions (ethnic minority; and/or income <¥8k; and/or ≤grade 6).

Statistics that reach the .05 level of significance are bolded.

#### Ethnic Inequalities and Urban/Rural Stratification (H1 and H3)

As shown in Table 2 (Model 3 and Model 4), there is an urban/rural stratification where the observed ethnic gap in functional limitations was mainly driven by rural participants (n = 8,662): rural ethnic minority was 44% more likely to have IADLs limitations than Han Chinese (OR = 1.44, 95%CI = 1.19–1.75); whereas this pattern was not statistically significant in the urban population, consistent with the bivariate analysis. To take a deeper look into the disaggregated data of ethnic disparities among rural participants, as shown in Table 3, the study found certain ethnic minority groups that had significantly elevated odds of having multiple IADLs limitations, even after adjusting for nine confounders. In the multinominal logistic regression analyses (Table 3), the odds of having ≥ 3 IADLs limitations were greater for Mongolians (OR = 5.36, 95% *CI*: 1.72–16.71), Tibetans (OR = 6.17, 95% *CI*: 3.23–11.81), Yi ethnic minority (OR = 3.32, 95% *CI*: 1.50–7.38) relative to Han majority Chinese. There were no significant differences in IADLs limitations for Miao ethnic minority, Buyi ethnic minority, and Manchu ethnic minority when compared to the Han Chinese.

#### Socioeconomic Inequalities and Urban/Rural Stratification (H2 and H3)

As shown in Table 2, a social class gradient in functional impairment stood out (see Model 1), especially among urban participants (see Model 3). There was a dose–response relationship between family income level and IADLs limitations. In other words, with decreasing income, the odds of reporting IADLs limitations significantly escalated, ranging from middle-income persons with 15% higher odds (OR = 1.34, 95% *CI*: 1.01–1.33) to individuals in the poorest bracket having 63% greater odds (OR = 1.63, 95% *CI*: 1.83–2.84) compared to the wealthiest respondents. There was a similar pattern between educational attainment and IADLs limitations. As education level decreased, a greater proportion of respondents reported having IADLs limitations (ORs range from 1.22 to 2.50), among whom the odds of having IADLs limitations in the lowest educational group rose more than two times (OR = 2.50, 95% *CI:* 2.05–3.06) in comparison to their peers in the highest educational bracket. As shown in Table 3, the income gradient was also visible in the odds of having ≥3 IADLs limitations (ORs range from 1.68 to 1.99) among the rural participants, whereas the education gradient was less pronounced in this outcome category.

#### Intersecting Social Vulnerabilities (H4)

**Figure 1.**
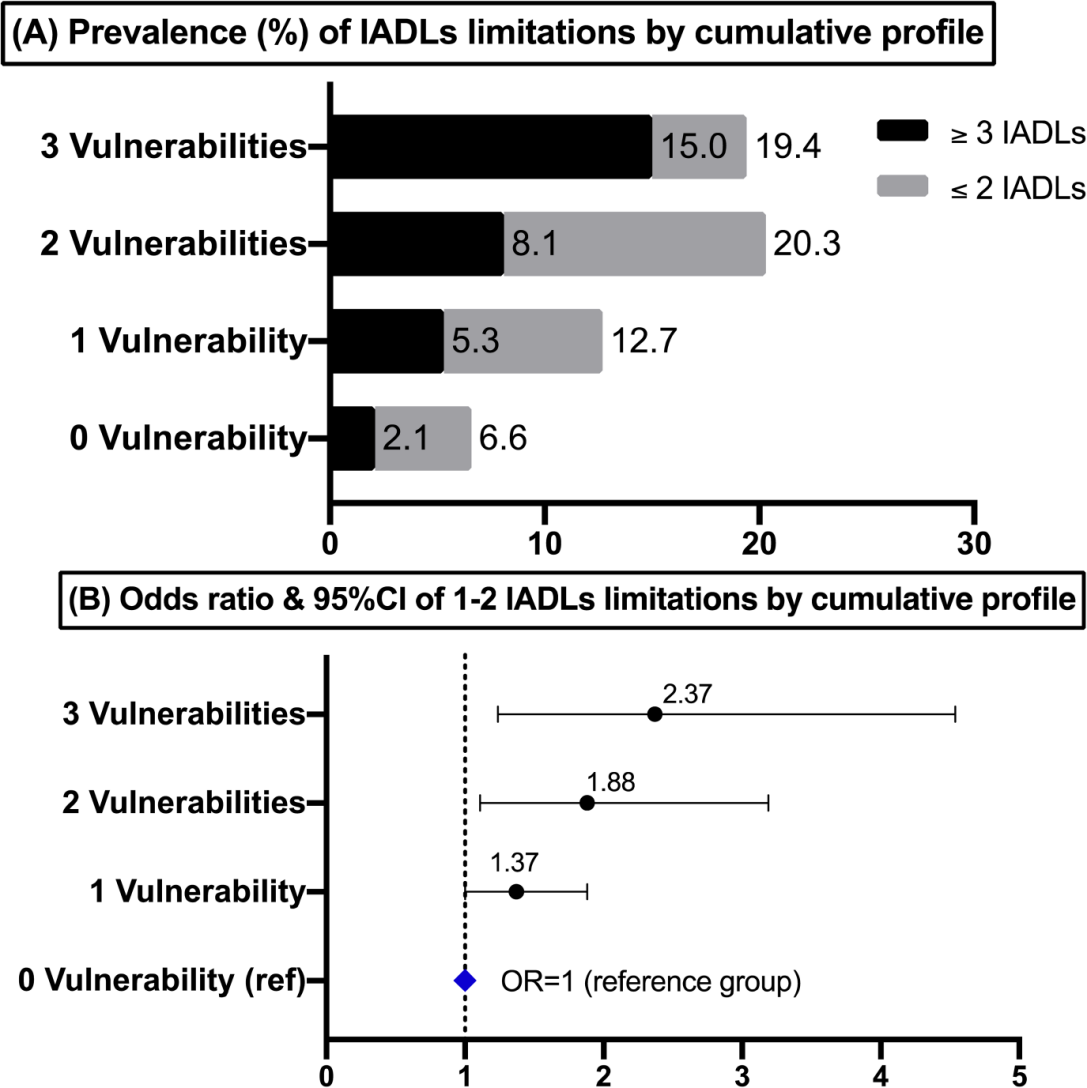
Prevalence (A) and adjusted odds ratios (ORs) (B) of IADLs limitations by the cumulative profile of ethnicity and social class among rural residents (n = 8,662) of CFPS 2018, persons aged ≥ 45 years. *Note*: ORs was adjusted for age, sex, marital status, retirement status, depression, self-rated health, health insurance, hospital visit, and social benefits. ORs were considered to be statistically significant when 95% *CI* did not overlap with 1.0. Cumulative profile = a sum of three social positions (ethnic minority Chinese; and/or income <¥8k; and/or ≤grade 6). CFPS = China Family Panel Studies; IADL = instrumental activities of daily living.

Table 3 demonstrates a clear dose–response relationship between cumulative social vulnerabilities and the possibility of having IADLs limitations among rural respondents (Figure 1 A & B). This association remained even after controlling for known covariates. Individuals who had more social vulnerabilities had higher odds of IADLs limitations. The largest jump in odds was observed in the transition from one to three social vulnerabilities. While one single marginalized status had 37% higher odds of having 1 to 2 IADLs limitations (OR = 1.37, 95% *CI*: 1.00–1.88), adding two social vulnerabilities more than doubled the likelihood (OR = 2.37, 95% *CI*: 1.24–4.54), compared to privileged individuals with no social vulnerability. Most importantly, as reported in Table 3, ethnic minority populations among the poorest socioeconomic strata (i.e., a combination of three vulnerable social conditions) had more than 5 times the odds of having ≥3 IADLs limitations relative to their Han Chinese peers with moderate to high socioeconomic standings (OR = 5.51, 95%CI: 1.93–15.71).

## Discussion

The current investigation is novel as it comprehensively examined the prevalence of IADLs disability among eight ethnic groups of Chinese and its relationship between three sources of social locations (i.e., ethnicity, income, and education) in China. It is the first of its kind investigation documenting ethnic disparities (i.e., Han-minority difference) in physical disability and the intersecting effect of ethnicity, income, and education on functional health disparities among middle-aged and older Chinese. These relationships could shed light on who may have a higher susceptibility to the development of physical disability in later life. The findings reveal that functional impairment is more prevalent among marginalized people who are ethnic minorities in rural China, urban elderly people with less educational attainment, and those living in low-income households in comparison to those who are Han Chinese, more educated, and/or more affluent. The results of this research indicate that socioeconomic and ethnic inequities in functional health exist in China as they do in industrialized countries such as the United Kingdom (Choi et al., 2020) and developing countries such as Turkey (Çakmur, 2015), Mexico (Smith & Goldman, 2007), and Brazil (Nóbrega et al., 2021). Taken together, these findings have important implications for future research, health promotion strategies, and clinical interventions among ethnic minority older adults in rural areas.

The novel assessment in this study has found nuanced ethnic inequalities in IADLs disabilities in rural China (supporting ethnic inequalities Hypothesis H1). The results indicated that certain ethnic minority elderly living in rural areas, particularly Mongolians, Tibetans, and Yi ethnic groups, had persistent disadvantages in IADLs disabilities even after adjusting for the independent effects of demographics, social class, and other health-enabling factors. Interestingly, Mongolians, Tibetans, and Yi ethnic groups were mainly residing at high-altitude mountainous areas in China (e.g., Yunnan–Guizhou Plateau), and they have adapted their livelihoods to the unique highland environment, such as diminished atmospheric pressure, highland climate, and exposure to sun radiations. The identified patterns of greater risks in IADLs disabilities among these three groups of highlanders may suggest that the highland habitat, including hypoxia and restricted environmental resources, could accelerate the human aging process (Matsubayashi et al., 2009). In addition, the current sample showed that ethnic minorities were more likely to be single or experienced widowhood in later life and, therefore, more prone to inadequate informal care support. These ethnic inequalities observed are consistent with previous research on compromised health conditions among non-Han Chinese populations, such as the higher prevalence of pain (Wang et al., 2013), obesity (Hui-Fang et al., 2019), and hypertension (Li et al., 2012). However, our finding contrasts with previous studies documenting the protective effect of ethnic minority status on ADLs disability among oldest-old in China (Gu & Xu, 2007; Gu & Zeng, 2004), partly due to age differences in the analytic samples where the oldest-old sample was at greater risk of selective survival with ethnic minorities having higher mortality rate.

The social class gradient in IADLs disability confirms the socioeconomic inequalities hypothesis (H2) and is in keeping with prior gerontological studies for Chinese older adults (Feng et al., 2013; Liu et al., 2020). A robust inverse association with a dose–response relationship between education attainment and IADLs limitations among urban older adults resonates with the linear trend of functional status decline predicted by baseline socioeconomic factors in prior longitudinal studies (Beydoun & Popkin, 2005). The urban–rural stratification hypothesis (H3) was validated empirically as the findings illustrate the effect modification by the urban–rural residency on the prevalence of IADLs disability in China. As such, this study extends existing health research on the urban–rural divide and the hukou registration system (Zhu & Österle, 2017; Zimmer & Kwong, 2004). This result highlights the vulnerability of rural ethnic minority older populations whose environmental and social conditions are still challenging (e.g., lack of accessible health services, insufficient health insurance coverage) that may lead to delayed diagnosis and undertreatment of chronic diseases with disabling impact.

Another contribution of the study is that it is the first attempt to systematically explore the associations between intersecting profiles of social vulnerabilities and IADLs disability in the Chinese populations. Supporting the intersecting inequalities hypothesis (H4), a gradient effect of cumulative social vulnerabilities on functional health disparities was found when ethnic minority identity and unfavorable socioeconomic positions were combined, which substantiates the utility of intersectionality theory as an analytical tool for examining health inequalities in China (Harari & Lee, 2021). This result underscores the necessity to move beyond a simplistic focus on one unitary category of difference in examining late-life disability (Roy et al., 2020). The present study presents solid evidence by arguing that multiple marginalization, such as those experienced by low-educated, low-income ethnic minority older adults residing in rural villages, were mutually constituted and reinforced in producing physical impairment at older age. Theoretically, it adds to the emerging scholarship that bridges structural, intersectional, and health equity lenses to aging research in the Global South (Roy et al., 2020; Warner & Brown, 2011). For policymakers and geriatric clinicians, this finding urgently calls for targeted initiatives to enhance LTC services for ethnic minority older adults who are living in low-income households to cope with environmental demands in rural mountainous regions.

## Strengths and Limitations

This population-based study has several strengths, including its large sample size, its ability to include measures of intersecting social positions, its pragmatic categorization of diverse ethnic minority Chinese subgroups, and its attention to the clustering of functional limitations in the aging populations. However, several methodological flaws and biases limited the generalizability of this study. First, relatively small sample sizes (n < 50) of certain minority groups (e.g., Mongolians and Buyi ethnicity in the rural sample) have weakened the statistical power; thus, estimates and confidence intervals for these two groups may contain instability and should be interpreted with caution. Second, the cross-sectional nature of the analysis prohibits the examination of causality. Third, the statistical models will always have the risk of “residual confounding”, resulting from unobserved characteristics that are not included in the analyses. Fourth, this study relied on self-reported survey data and therefore is susceptible to recall bias, misreporting, and misclassification. For example, the IADLs scale is a self-administered test rather than the actual demonstration of the functional task, which may lead to either an overestimation or underestimation of one's functional capacity. The CFPS dataset did not collect the information about basic ADLs that could measure more severe forms of functional impairment (e.g., difficulty toileting, dressing, or bathing). Future studies could investigate functional health disparities of ethnic heterogeneity among Chinese older populations by contrasting self-rated and performance-based physical health assessments, including IADLs, ADLs, and functional mobility (Purser et al., 2012). Lastly, the CFPS excludes individuals residing in several ethnic autonomous regions (e.g., Tibet province), which would result in an underestimate of the disability prevalence among ethnic minority groups.

## Conclusion and Policy Implications

This nationwide study has demonstrated inequalities by ethnicity, income education, and rural–urban residency in the prevalence of IADLs disability among noninstitutionalized middle-aged and older Chinese. Such functional health inequalities are potentially amenable to policy beyond the scope of the traditional health sector. Social policies that redistribute resources could address this problem, for example, by expanding social welfare programs (e.g., old-age pension, unemployment insurance, public housing, and the subsidy of prescription drugs) for marginalized older adults, particularly those living in rural mountainous areas. Notably, China has achieved significant progress in poverty reduction by lifting more than 68 million people living in rural regions out of poverty via cross-regional Pairing Aid Programme - a system that assigns economically advanced entities (i.e., provinces and state-owned enterprises) in the eastern coastal urban areas to build partnership with and provide financial assistance to an underdeveloped counterpart in rural remote regions of China (Lin, 2022). Another intervention to resolve the endogenous poverty trap in mountainous areas lies in the anti-poverty relocation and settlement program (Li et al., 2021), through which 9.6 million rural villagers were resettled into publicly subsidized housing and brand-new neighborhoods with modern infrastructures. These anti-poverty policies could tackle upstream social inequities, reduce urban-rural disparities and enhance vulnerable individuals’ capacity to cope with day-to-day life challenges (Phelan & Link, 2005).

Another rapid approach lies in redirecting health-enhancing resources to people living in lower-income and ethnic minority communities. Since 2013, China has prioritized the integration of health care and LTC systems (“*yi-yang-jie-he”*) on the LTC policy agenda and has begun piloting social insurance LTC financing models in 15 cities (Feng et al., 2020; Zhu & Österle, 2017). Similar to many integrated care models for older adults (e.g., the Chronic Care Model) that have gained popularity in the Global North (Epping-Jordan et al., 2004), the *yi-yang-jie-he* policy aims to coordinate multiple levels of care across different settings, resolve service fragmentation and improve care quality for geriatric patients. To sum up, the findings of this national study suggest that, in order to achieve the Healthy China 2030 blueprints (Yang et al., 2018), equity should be placed at the center of all LTC policies to eliminate inequalities in responding to escalated LTC needs among ethnic minorities in rural China. Equity-driven initiatives involve the formulation of home and community-based services targeted for vulnerable older adults who are living in rural remote areas in China that are potentially left behind by the current health care system.
